# American Veterinary College

**Published:** 1899-07

**Authors:** 


### AMERICAN VETERINARY COLLEGE.

History is not written in a day. The record of a college not made in a year. Its growth is slow and its value to a state or country measured by its achievements over a period of many years of sunshine and shadow; through periods of prosperity and times of adversity. From the results of a quarter of a century's work the history of this institution may now be reckoned and considered. With to-day more than six hundred of her offspring, making up a large quota of the American veterinary profession, among its numbers are to be found many of the most active members of the calling in every state and territory. Starting in 1875 on a sound basis, that of giving the best to be afforded her students in the line of a thorough and complete education, she has well equipped men for every demand and need that has arisen in the rapid progress of our land, and made some lines of veterinary sanitary work possible that could not have been maintained save by the scope of education given her students. The growth of other schools, the army, the experiment station, the agricultural colleges, the bureau of animal industry, the boards of health, the breeding farms, the field of commerce, international, national and state, have all felt the impulse of her influence and reaped the advantages derived by the recognition of her graduates, while every section of our own land, and many fields on foreign soil, basked in the security afforded by the skill of her sons, in the invested interests wrapped up in their live-stock possessions.

With an unsullied career in the march of higher education ever directed by one whose sincerity and devotion to the best interests of the profession stand unchallenged, she has marked out in the history of veterinary education in America a proud record. The younger institutions of our land with their greater resources, their grander possibilities, may well study the history of this institution, and invoke to their greater responsibilities the determined spirit, the unflinching courage, the resistance to temptation, that have made possible this tribute to the American Veterinary College. All honor to those who have directed her affairs, all praise to those who have sustained her career, and well wishes for her future and the welfare of her sons!

